# Phylogenetic and Environmental Insights Into the Biogeography of the Western Blacknose Dace, *Rhinichthys obtusus*

**DOI:** 10.1002/ece3.73849

**Published:** 2026-06-16

**Authors:** Adelina Rodriguez, Timothy S. Earley, Samuel Taylor, Thomas E. Dowling, Phillip M. Harris, Jeremy Wright, Antonio Machado‐Allison, Barry Chernoff

**Affiliations:** ^1^ Earth & Environmental Sciences Wesleyan University Middletown Connecticut USA; ^2^ Bailey College of the Environment Wesleyan University Middletown Connecticut USA; ^3^ Department of Biology Wesleyan University Middletown Connecticut USA; ^4^ Biological Sciences Wayne State University Detroit Michigan USA; ^5^ Department of Biological Sciences University of Alabama Tuscaloosa Alabama USA; ^6^ New York State Museum Albany New York USA

**Keywords:** biogeography, freshwater fish, glacial refugia, mitochondrial genomics, Pleistocene glaciation, species distribution modeling

## Abstract

Phylogeographical patterns in North American freshwater fish are linked to the evolution of drainage configurations and have been shaped by the Pleistocene glaciations, especially throughout the Mississippi River drainage and eastern North America. This study investigated the recent phylogeographical history of *Rhinichthys obtusus* , Western Blacknose Dace, by integrating mitochondrial genomic DNA phylogenetics with geologic and paleoclimate data. This monophyletic species, recently determined to be genetically distinct from its sister species *Rhinichthys atratulus* , represents an important model to study the phylogeographic history of a North American stream fish in depth. Maximum‐Likelihood and Bayesian analysis trees based on full consensus mitochondrial genomes resolved three major clades of *R. obtusus* : An Eastern Great Lakes clade, a Southeast clade, and an Upper Midwest clade. Our phylogenetic analyses suggested these three clades diverged 2–1.5 million years ago during the Pre‐Illinoian glaciations, when the advancing ice sheet altered river systems and landscapes and broke up populations, constricting ancestral populations of *R. obtusus* to refugia in the Interior and Eastern Highland regions. Based on present and historical MaxEnt species distribution models as well as geologic evidence, recolonization into the Eastern Great Lakes and Upper Midwest regions may have primarily occurred from around 15 to 12 thousand years ago as glaciers retreated, habitat suitability increased, and river connections appeared. Our findings contribute to an understanding of the evolutionary history of *Rhinichthys*, and to a broader discussion of freshwater fish phylogeography in North America.

## Graphical abstract

Pleistocene glaciations have shaped freshwater fish evolution and distribution patterns across North America. This study investigated the phylogeographical history of the Western Blacknose Dace ( *Rhinichthys obtusus* ) using mitochondrial genomic data, revealing three major clades that diverged 2–1.5 million years ago during Pleistocene glaciations when ice sheets fragmented populations and pushed them into refugia in the Mississippi River, Tennessee River, and Mobile Bay drainage basins. Phylogenetic and species distribution modeling analyses suggest these populations recolonized the Eastern Great Lakes and Upper Midwest regions approximately 15–12 thousand years ago following glacial retreat.

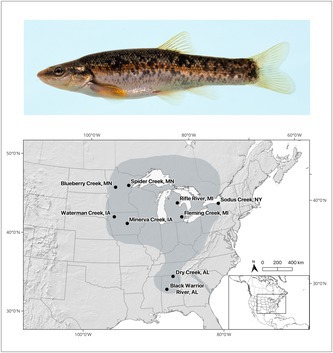

## 1 Introduction

Geographic and genetic diversity of freshwater fish in North America has been the focus of extensive research over the years. This is especially true for fish in the southeastern United States and throughout the Mississippi River Basin, where species richness and diversity are highest (Smith et al. 2010; Jenkins et al. 2015). A key question of interest is how historical climate and geologic events, such as the Pleistocene glaciations, have facilitated the increase in genetic variation within and among species, and the spread of such diversity throughout the continent. The field of comparative phylogeography has made it possible to study this by integrating historical biogeography with population genomics and phylogenetics (Avise et al. 1987; Beheregaray 2008; Vaux et al. 2023). It is well known that freshwater fish phylogeography aligns closely with landscape evolution (Hocutt and Wiley 1986; Mayden 1988; Near et al. 2001; Berendzen et al. 2003; Soltis et al. 2006; Bossu et al. 2013; Waters et al. 2020). Therefore, this study focused on utilizing geologic evidence and paleoclimate data to better understand the role of the Pleistocene glaciations and related landscape changes in shaping the phylogeographical history of *Rhinichthys obtusus* Agassiz (1854), Western Blacknose Dace.

**FIGURE 1:**
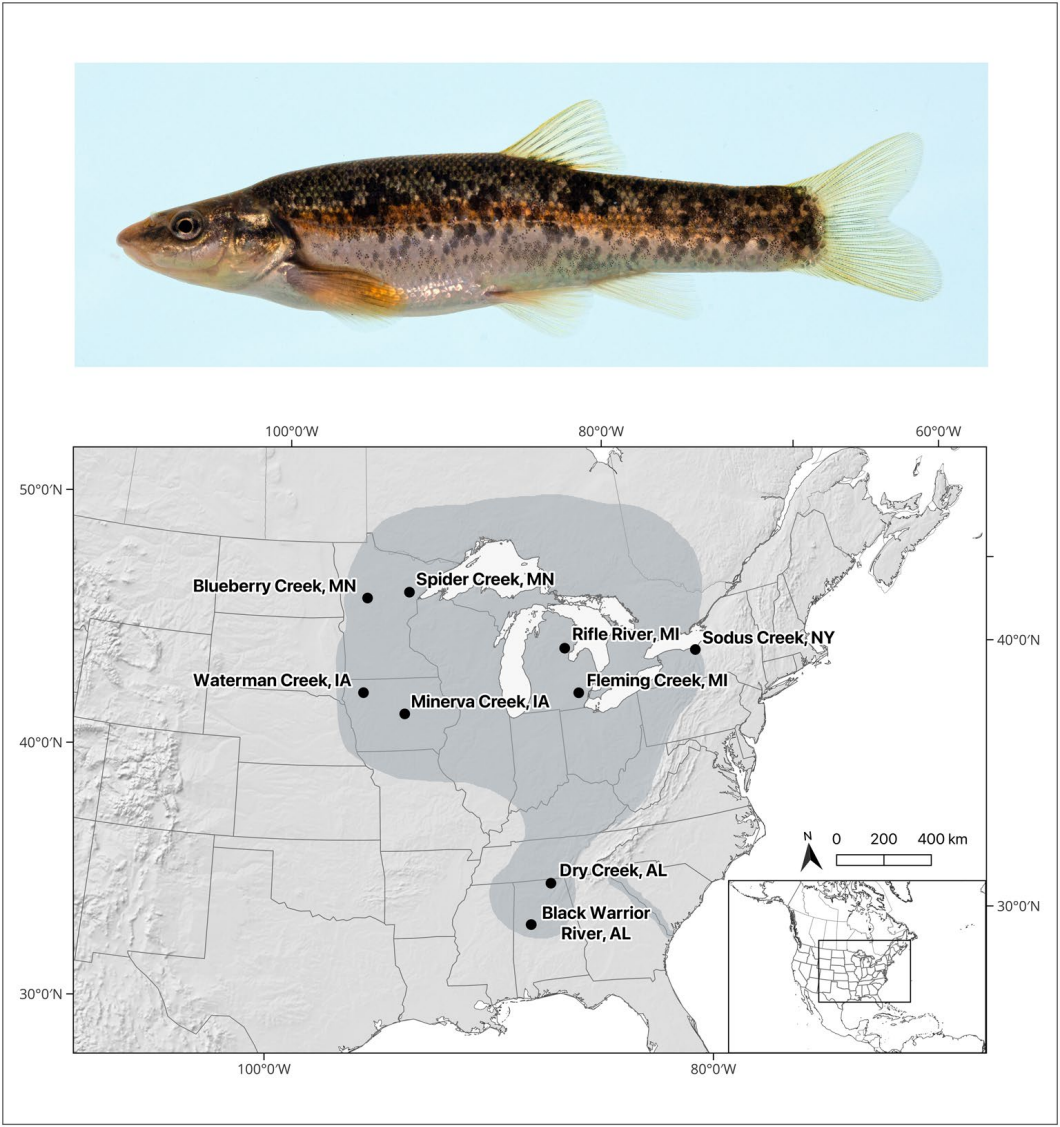
Top: Western Blacknose Dace, *Rhinichthys obtusus* , individual collected from Waterman Creek, IA. Bottom: Map showing nine study sample localities in Michigan, New York, Minnesota, Iowa, and Alabama, USA, North America. The modern distribution of *R. obtusus* (blue‐gray shaded area) was estimated based on Kraczkowski and Chernoff (2014) and MaxEnt modeling (v. 3.4.4; Phillips et al. 2024).

*R. obtusus* is an example of a freshwater minnow species with disjunct populations that range from Hudson Bay drainages in the North to the southeast Mobile Bay drainage (Figure 1). The genus *Rhinichthys* comprises a monophyletic group of eight species of North American, riffle‐dwelling minnows (size < 150 mm SL) of the family Leuciscidae, residing in small to medium‐sized streams across North America (Woodman 1992; Kraczkowski and Chernoff 2014; Smith et al. 2017; Schönhuth et al. 2018; Earley et al. 2024). *R. obtusus* was only recently characterized as genetically distinct from sister species *Rhinichthys atratulus* Herman (1804) based on two mitochondrial genes and nine microsatellite loci sampled from more than 800 individuals of the *R. atratulus‐R. obtusus* complex (Kraczkowski and Chernoff 2014). *R. obtusus* primarily inhabits Mississippi drainages to the west of the Appalachian Mountains, including the Tennessee and Cumberland Rivers, the Upper Midwest region, and the Ohio River. Populations have also been found in tributaries of Hudson Bay, the Great Lakes and the St. Lawrence River; in Atlantic Slope drainages of the Santee system and the Savannah River basin in North and South Carolina; and in the Upper Coosa and Black Warrior rivers in the Mobile Bay basin (Kraczkowski and Chernoff 2014). The biogeographic history of *R. obtusus* has yet to be studied in depth.

Previous studies on the phylogenetics and population dynamics of *R. obtusus* and sister species *R. atratulus* found distinct groupings among individuals (Kraczkowski and Chernoff 2014; Ejaz 2023; Rodriguez 2025). Kraczkowski and Chernoff (2014) found potential sub‐clades of *R. obtusus* in separate drainages of the Great Lakes, Mississippi, and Atlantic Slope. Ejaz (2023) and Rodriguez et al. (n.d.) utilized high‐throughput sequencing to characterize genomic variants across six populations within the *R. atratulus* complex. In these two studies, analyses of single nucleotide polymorphisms (SNPs), small insertion‐deletions (indels), and large structural variants (SVs) revealed that *R. obtusus* populations from Michigan and Alabama were genomically different but closely related, corresponding to prominent freshwater fish phylogeographic patterns of post‐glacial recolonization into the Great Lakes Region from southern refugia (Mayden 1988). In the present study, we expanded upon these previous works in population genomics of *R. obtusus* by including additional Upper Midwest populations as well as the reference genome of its sister species, *R. atratulus* . Intraspecific variation present within *R. obtusus* prompts further investigation into the role of dispersal and vicariance in setting the modern distribution of the species across the Eastern Great Lakes, the Southeast, and the Upper Midwest region.

In this study, we examined the phylogeography of *R. obtusus* using whole mitochondrial genome sequences from 86 individuals in nine populations in Minnesota, Iowa, Michigan, New York, and Alabama, representing the outer extent of its geographic range (Figure 1). Relative to nuclear DNA (nuDNA), mitochondrial DNA (mtDNA) evolves quickly, undergoes fast lineage sorting, is maternally inherited, and is abundantly available for molecular analyses (Moore 1995; Palumbi et al. 2001; Rubinoff and Holland 2005; Allio et al. 2017). mtDNA trees can therefore resolve phylogenetic relationships between closely related species and intraspecific lineages, such as clades within *R. obtusus* (Moore 1995; Bossu and Near 2009; Bryson et al. 2014; Chen et al. 2022). Furthermore, utilizing full mitochondrial genomes rather than select genes may provide even more robust phylogenies (Zhang et al. 2021; Chen et al. 2022).

We focused on three main objectives in this study. We first constructed two separate phylogenetic trees from the full mitochondrial genome to assess relationships (also known as phylomitogenomics); one Maximum‐Likelihood tree and one Bayesian tree to estimate divergence times between clades. Second, we modeled potential habitat suitability for *R. obtusus* in North America to illustrate historical distributions in several time increments after the Last Glacial Maximum (LGM). Third, we reconstructed and mapped dispersal and vicariance events associated with *R. obtusus* distributions and recolonization into northern regions. An additional key objective was to compare our findings and hypotheses with the phylogeographic patterns of other freshwater fish species in North America.

## 2 Methods

### 2.1 Sample Collection and Short‐Read Sequence Generation

We first generated whole genome sequences for *R. obtusus* individuals in order to isolate mitochondrial genomes (mitogenomes) through mapping to a reference mitogenome. The following sections detail our sample collection and whole genome sequencing protocols, describe the generation of the reference genome, and outline our process for mitogenome isolation from *R. obtusus* specimens.

To generate whole genome sequences, we collected DNA data from 86 individual fish from nine populations of *Rhinichthys obtusus* (Figure 1). 29 fish specimens (9–10 per site) from Michigan and Alabama were obtained from Ejaz (2023). We collected an additional 57 specimens in 2024 from New York, Minnesota, and Iowa. The following is a list of the sampled localities including the number of specimens: (i) New York, Sodus Creek in Wayne County, (43.192° N, 76.912° W), number (*n*) = 10 individuals; (ii) Michigan, Fleming Creek in Washtenaw County, (42.317° N, 83.646° W), *n* = 9; (iii) Michigan, Rifle River in Ogemaw County, (44.179° N, 84.073° W), *n* = 10; (iv) Alabama, Dry Creek in Jackson County, (34.824° N, 86.306° W), *n* = 9; (v) Alabama, Black Warrior River in Jefferson County, (33.252° N, 87.461° W), *n* = 10; (vi) Iowa, Minerva Creek in Marshall County, (42.078° N, 93.188° W), *n* = 10; (vii) Iowa, Waterman Creek in O'Brian County, (42.968° N, 95.425° W), *n* = 10; (viii) Minnesota, Spider Creek in St. Louis County, (46.978° N, 92.680° W), *n* = 10; (ix) Minnesota, Blueberry Creek in Wadena County, (46.784° N, 95.149° W), *n* = 10. These geographic locations were selected to represent the outer edge of the known U.S. distribution of *R. obtusus* , including previously glaciated areas, therefore allowing us to test hypotheses regarding glacial refugia and post‐glacial recolonization. We were also able to test the hypothesis that populations of *R. obtusus* in these geographic regions contain intraspecific differentiation (Kraczkowski and Chernoff 2014; Ejaz 2023; Rodriguez 2025).

For whole genome sequencing, muscle tissue from specimens was sent to Novogene (Novogene Corporation Inc., Sacramento, CA, USA) for sequencing and production of short‐read libraries, using the NEBNext UltraTM DNA Library Prep Kit for Illumina (New England Biolabs, Ipswich, MA, USA) and 2 × 150 paired‐end sequenced on an Illumina platform (Illumina, San Diego, CA, USA). Sequencing runs produced 23.70 million reads and 3.23× coverage per sample (see Novogene mapping percentages for the additional 57 specimens in Supporting Phylogenetic Materials).

### 2.2 *Rhinichthys atratulus* Reference Mitochondrial Genome

The reference mitogenome was from Eastern Blacknose Dace, *Rhinichthys atratulus* , developed from the locality of an individual at Laurel Brook in Middletown, Connecticut. Because *R. atratulus* is the sister species to *R. obtusus* (Kraczkowski and Chernoff 2014; Earley et al. 2024), using *R. atratulus* as a reference resulted in high sequence mapping percentage, accurate alignments, and better variant calling. The Chernoff Lab created this full reference genome in 2024. A single whole fish specimen was sent to Cantata Genome Services (Cantata Bio LLC, Scotts Valley, CA, USA), and whole genomic DNA was extracted from muscle tissue and sequenced via PacBio circular consensus sequencing (Pacific Biosciences of California Inc., Menlo Park, CA, USA). The resulting reference genome is 1.11 Gb in length; composed of 25 chromosomes, a mitogenome, and 560 unplaced scaffolds; has an N50 value of 40.55 Mb, and a complete BUSCO Actinopterygii score of 99.0%. The *R. atratulus* mitogenome was then isolated from the de novo assembly using MINIMAP2 (v. 2.26; Li 2018) by aligning against the closely related *Rhinichthys cataractae* mitogenome and extracting the aligned scaffold using ‘seqtk’ (v. 1.3; Li 2012), as outlined in Earley et al. (2024).

### 2.3 *Rhinichthys obtusus* Mitochondrial Genome Isolation

Mitogenomes were retrieved from cleaned genomic short‐reads of our 86 sampled individuals. For each individual, we aligned paired‐end mitochondrial reads against the *R. atratulus* reference mitochondrial genome described above (Earley et al. 2024), then merged, sorted, and indexed subsequent .bam files using BWA (v. 0.7.8; Li and Durbin 2009), GATK suite (v. 4.4.0.0; Van der Auwera and O'Connor 2020), and SAMtools (v. 1.20, Danecek et al. 2021; Li et al. 2009). Consensus sequences (sequences generated by aligning multiple sequences and identifying the most probable nucleotide at each position based on frequency, read quality, and mapping quality) were then built from all individuals in each population with SAMtools. Whole mitogenomes were used with inclusion of the D‐loop and third codon positions. We chose to generate and analyze consensus sequences rather than individual sequences in order to gain an understanding of population‐level differentiation, as opposed to variation among individuals. Additional mitochondrial genomes were downloaded from NCBI for outgroup species to build phylogenetic trees (accession numbers are included in Supporting Phylogenetic Materials). We used MAFFT software (v. 7; Katoh and Standley 2013) via the Galaxy open‐source platform (The Galaxy Community 2024) to align all consensus sequences, including *R. obtusus* populations and outgroup species, into a single fasta file for analysis.

### 2.4 Phylogenetic Analysis of Mitochondrial Genomes

#### 2.4.1 Maximum‐Likelihood Phylogenetic Tree

We constructed a Maximum‐Likelihood tree using RAxML (v. 1.2.2; Stamatakis 2014; Kozlov et al. 2019) from consensus mitochondrial genomes for nine *R. obtusus* populations and individual *R. atratulus, R. osculus*, and *R. cataractae* . The TIM3 + G4 substitution model was tested using MODELTEST‐NG gui application (v. 0.1.7; Flouri et al. 2014; Darriba et al. 2020) and applied, with data partitioned by all three codon positions for protein‐coding genes. Node support was assessed using 3000 bootstrap replicates from 10 starting trees. Final trees were visualized using the FIGTREE application (v. 1.4.4; Rambaut 2010). We chose to build our mitogenome trees using data partitioned by all three codon positions because we analyzed closely‐related populations. As demonstrated by Earley et al. (2024), the third codon position effectively resolves closely‐related branches with high support for Pogonichthyine species that included *Rhinichthys*.

#### 2.4.2 Bayesian Divergence Time Estimation

Divergence times were estimated in BEAST2 (v. 2.7.7; Drummond and Rambaut 2007; Bouckaert et al. 2019) for mitochondrial consensus sequences for nine *R. obtusus* populations, individual *R. atratulus, R. osculus*, and *R. cataractae*, and selected outgroup species from the family Leuciscidae (see accession numbers in Supporting Phylogenetic Materials). Input XML files were generated with BEAUti (v. 2.7.7; Bouckaert et al. 2019). The TIM3 + G4 site substitution model was applied with data partitioned by all three codon positions for protein‐coding genes, a relaxed lognormal clock to allow for rate variation (Drummond et al. 2006; Lepage et al. 2007; Wertheim et al. 2010), and a constant population size coalescent model (Kingman 1982; Drummond et al. 2002; Gill et al. 2013).

Divergence estimates were constrained using three fossil‐calibrated MRCA priors from the literature: *R. osculus* and *R. obtusus/R. cataractae* (4.83–7.81 ma, Smith and Dowling 2008; Kim and Conway 2014), *R. cataractae* and *R. atratulus* (6.50–10.87 ma, Kim and Conway 2014), and *R. cataractae* and *Oregonichthys crameri* , Oregon Chub (9.06–13.06 ma, Kim and Conway 2014). Uniform distributions were set for each date range. When running BEAST2, MCMC chains were run for 10 million generations and sampled every 1000 steps. Posterior trees were combined in LOGCOMBINER (v. 1.10; Bouckaert et al. 2019) and summarized in TREEANNOTATOR (v. 1.10; Bouckaert et al. 2019) with a 15% burn‐in. The final tree with node ages was visualized on FIGTREE (v. 1.4.4; Rambaut 2010) and annotated with a geologic time scale using the ‘ggtree’ package in R (v. 1.14.6; Yu et al. 2017).

### 2.5 Modern and Historical Species Distribution Modeling

Species distribution models (SDMs) aim to predict full geographic ranges of species across time, benefiting evolutionary biology studies and helping to improve our understanding of shifting habitats (Elith et al. 2006; Austin 2007; Elith and Leathwick 2009; Zurell et al. 2020). We modeled current and historical distributions of *R. obtusus* using MaxEnt (v. 3.4.4; Phillips et al. 2006; Phillips et al. 2024), which utilizes a maximum entropy algorithm with presence‐only species data points and environmental raster variables (Phillips et al. 2006; Merow et al. 2013).

#### 2.5.1 Species Presence Data and Climate Predictor Variables

Occurrence records (10,702 latitude, longitude points) were compiled from GBIF (GBIF.org 2024a, 2024b, 2024c) for data points listed under *R. obtusus, R. atratulus*, and *R. atratulus obtusus* , to account for taxonomic uncertainty. Because members of the *R. atratulus* complex ( *R. atratulus* and *R. obtusus* ) reside in similar riffle environments, the presence data and models reflected overall suitable habitat, but did not delineate the range of *R. obtusus* precisely.

All 19 standard bioclimatic variables at 1 km resolution were obtained from the CHELSA database for current and historical data (https://chelsa‐climate.org). Modern climate conditions were derived from the ERA‐Interim global reanalysis and averaged from 1981 to 2010 (v. 2.1; Karger et al. 2017, 2020). Paleoclimate data were derived from the CCSM3 TraCE‐21 k climate model (Liu et al. 2009; He 2011; Carlson et al. 2012) and downloaded for time steps spanning 21.5 ka to 9 ka (v. 1.2; Karger et al. 2020; Karger, Lange, et al. 2023; Karger, Nobis, et al. 2023).

We then reduced correlation and multicollinearity of the climate variables to avoid singularities and overfitting of the models (Dormann et al. 2013; Brun et al. 2020; Sillero et al. 2021). Variables with variance inflation factors (VIF) > 10 were eliminated in a stepwise approach using the R package ‘usdm’ with the function ‘vifcor’ (v. 2.1–7; Naimi et al. 2014, Sillero et al. 2021). The final set of variables included Bio5 (max temperature of warmest month), Bio7 (temperature annual range), Bio12 (annual precipitation), Bio15 (precipitation seasonality), and Bio17 (precipitation of the driest quarter). A digital elevation model at 30 arc‐seconds resolution from Global Multi‐resolution Terrain Elevation Data 2010 (Danielson and Gesch 2010) was also included (Table 1). All raster layers were clipped to the extent of North America and were projected into WGS 84 using ArcGIS Pro (Esri 2025).

**TABLE 1:** Climate predictor variables from the CHELSA online database compiled for modeling *R. obtusus* current and historical distributions. All CHELSA raster layers have a spatial resolution of 1 km. The digital elevation model (dem) has a resolution of 30 arc‐seconds (approximately 1 km).

Variable	Description	Explanation	Units
Bio 5	Maximum temperature of warmest month	The highest temperature of any monthly daily mean maximum temperature.	°C
Bio 7	Temperature annual range	The difference between the Maximum Temperature of Warmest month and the Minimum Temperature of Coldest month.	°C
Bio 12	Annual precipitation	Accumulated precipitation amounts over 1 year.	kg m^−2^ year^−1^
Bio 15	Precipitation seasonality (coefficient of variation)	The Coefficient of Variation is the standard deviation of the monthly precipitation estimates expressed as a percentage of the mean of those estimates (i.e., the annual mean).	unitless
Bio 17	Precipitation of the driest quarter	The driest quarter of the year is determined (to the nearest month).	kg m^−2^ quarter^−1^
Elevation	Surface elevation above sea level	Global topographic elevation model designated as GMTED2010 at a horizontal resolution of 30 arc‐seconds for the entire Earth.	m

#### 2.5.2 MaxEnt Modeling Analysis

MaxEnt models were run with auto features, 500 iterations, and 10 cross‐validation replicates using subsets of 95% training and 5% testing. A regularization multiplier value of 0.5 was applied after evaluating model performances using regularization multiplier value settings from 0.5 to 5 in 0.5 increments. A maximum of 10,000 background points were used (Barbet‐Massin et al. 2012; Kanagaraj et al. 2013; Iturbide et al. 2015). Outputs were in clog‐log format, with probability of occurrence estimates ranging from 0 to 1. Model performance was assessed using AUC from receiver operating characteristics (ROC) curves, with values > 0.5 indicating good performance and > 0.9 indicating very high performance (Elith 2000; Elith et al. 2006; Phillips et al. 2017; Anderson and Gonzalez 2011; Jiang et al. 2018). Additionally, omission rates, the percentages of known presence points incorrectly classified by the model as unsuitable habitat, were reported for (1) minimum training presence (MTP) omission rate (based on all data, ideally close to 0% omission) and (2) 10th percentile training presence omission rate (omits the lowest 10% of training presence points, ideally close to 10%) (Bao et al. 2022). High omission rates indicate model overfitting, and a large difference between training and test values indicate poor model performance (Bao et al. 2022; Yousaf et al. 2022).

Geospatial model results were generated in QGIS (v. 3.40.2; http://qgis.org 2025). All maps included base layers and cartographic boundary shapefiles from Natural Earth Data (http://naturalearthdata.com), as well as paleogeographic shapefiles from literature: time series of Laurentide Ice Sheet (LIS) extents (Dalton et al. 2023), permafrost extent at the Last Glacial Maximum (LGM) (Lindgren et al. 2016), and paleodrainage boundary lines (Wickert 2016; updated 2025, pers. comm.).

## 3 Results

### 3.1 *R. obtusus* Phylogenetics and Divergence Time Estimates

#### 3.1.1 RAxML Maximum‐Likelihood Tree

**FIGURE 2:**
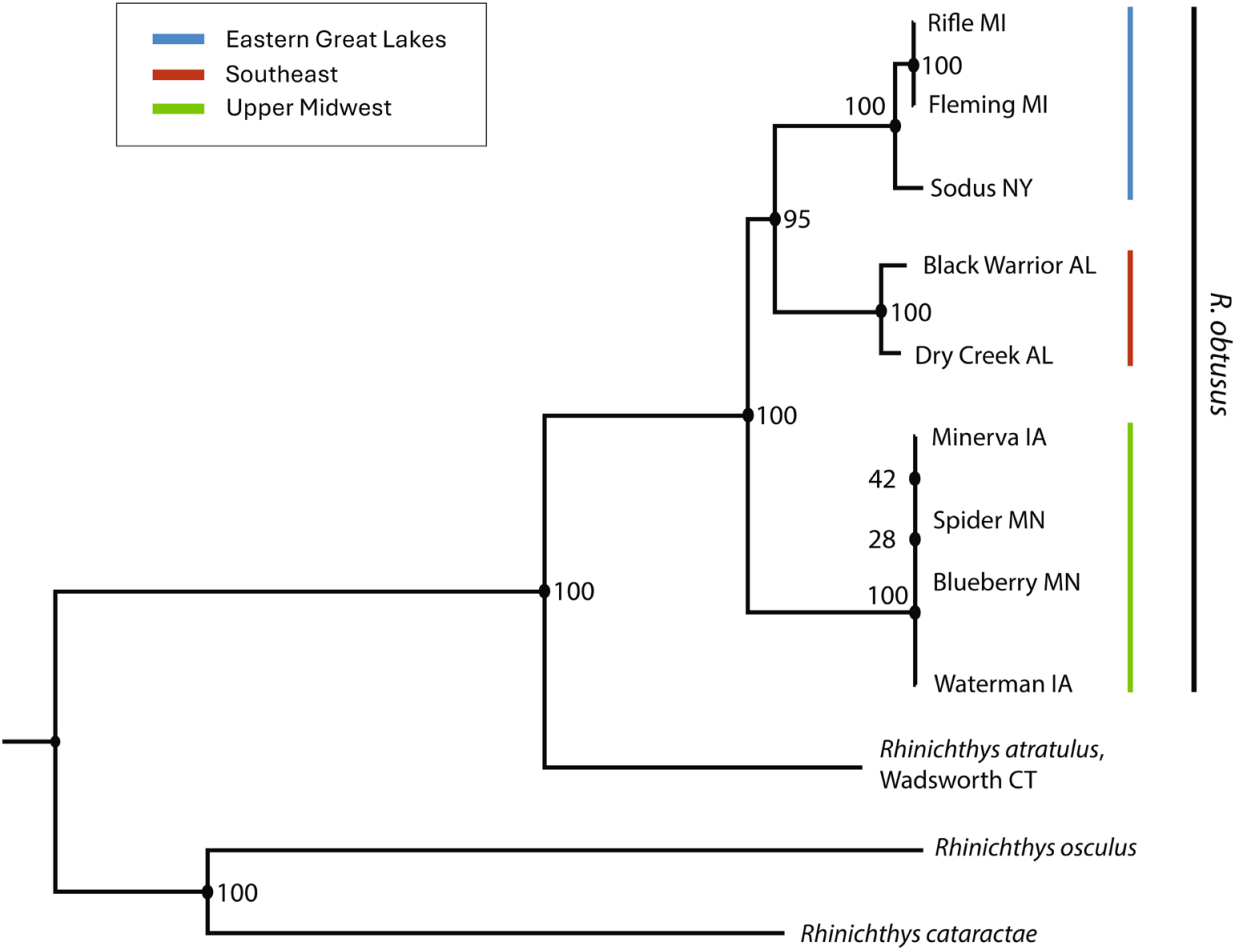
Maximum‐Likelihood tree based on aligned mitochondrial consensus genomes for each population plus three related *Rhinichthys* groups, created using RAxML with a TIM3 + G4 nucleotide substitution model (v. 1.2.2; Stamatakis 2014). Bootstrap values were calculated for each node.

RAxML analysis resolved seven out of nine nodes with strong bootstrap (BS) support (> 90%) (Figure 2). The tree was rooted to the outgroup *R. cataractae* (Earley et al. 2024), which predictably formed a monophyletic group with *R. osculus* (Smith and Dowling 2008; Kim and Conway 2014). *Rhinichthys atratulus* from Laurel Brook in Connecticut was sister to the *R. obtusus* clade with 100% BS support. Within the *R. obtusus* clade, three monophyletic groups were identified with full support: Michigan and New York populations (A, Eastern Great Lakes clade), Alabama populations (B, Southeast clade), and Minnesota and Iowa populations (C, Upper Midwest clade). The Eastern Great Lakes and Alabama clades were sister groups, forming a monophyletic group outside the Upper Midwest clade.

Relationships among populations in the Upper Midwest clade were poorly resolved due to minimal mitochondrial sequence divergence. Minerva IA and Spider MN grouped together (42% BS support) with Blueberry MN placed outside of them (28% BS) and Waterman IA outside those three (100% BS). These results conflicted with the Bayesian analysis, which grouped Minerva IA and Blueberry MN together, with Spider MN (Lake Superior drainage) placed on the outside.

#### 3.1.2 BEAST2 Bayesian Analysis Tree With Divergence Time Estimates

**FIGURE 3:**
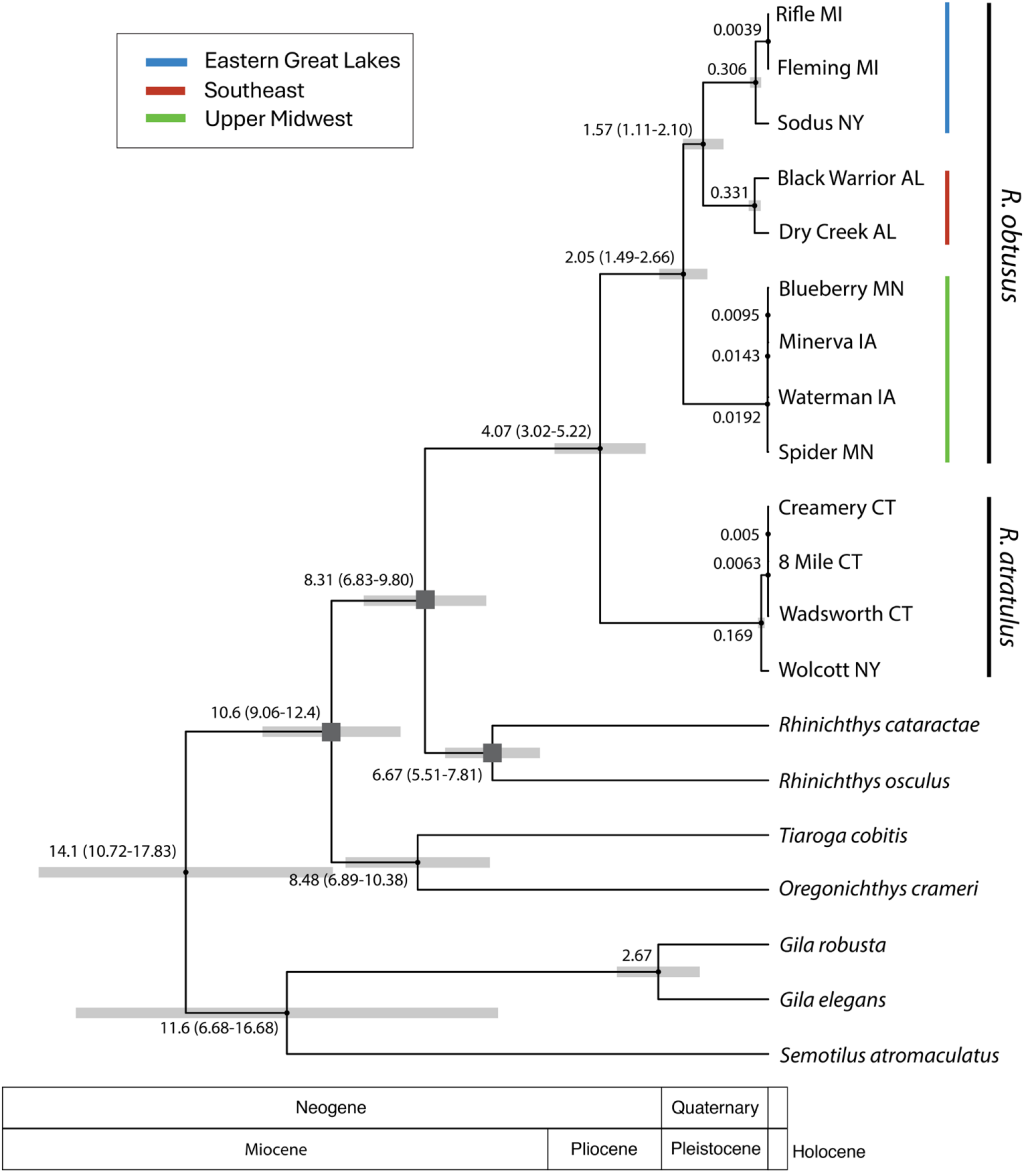
Bayesian phylogenetic tree including timing of divergence events (numbers are in millions of years) of *R. obtusus* populations and several outgroup species, produced in BEAST2 (v. 2.7.7; Bouckaert et al. 2019). The three dark gray squares mark the points calibrated by fossil date ranges (Smith and Dowling 2008; Kim and Conway 2014). The light gray bars represent 95% highest posterior density (HPD) for node age estimates. The analysis used a TIM3 + G4 nucleotide substitution model, a relaxed molecular clock with a lognormal distribution, and a coalescent constant population size model.

Bayesian analysis with BEAST2 supported the same three major clades within *R. obtusus* and calculated potential divergence times (Figure 3). The Upper Midwest clade diverged from the Eastern Great Lakes/Southeast populations ~1.5–2.7 ma (1.8%–3.2% divergence), while the Eastern Great Lakes and Southeast clades split ~1.1–2.1 ma (1.5%–3.0%). Divergence between *R. obtusus* and *R. atratulus* was estimated at ~3.0–5.2 ma (3.7%–6.4%) in the late‐Pliocene, and their divergence from *R. cataractae* and *R. osculus* occurred ~6.8–9.8 ma (14.5%–20.7%), consistent with fossil dates supplied to the model (Smith and Dowling 2008; Kim and Conway 2014). The split between *R. cataractae* and *R. osculus* (5.5–7.8 ma, 6.5%–9.2%) agreed with the 4.5–9.2 ma time range of Hoekzema and Sidlauskas (2014). The clade containing *Tiaroga cobitis* and *O. crameri* putatively split from *Rhinichthys* groups ~9.1–12.4 ma (10.6%–14.6%), and the clade containing *Gila robusta, Gila elegans*, and *Semotilus atromaculatus* split from *Rhinichthys* ~10.7–17.8 ma (12.6%–20.9%).

Within major clades of *R. obtusus* , the model indicated that populations diverged in the mid‐ to late‐Pleistocene. According to the model, in the Eastern Great Lakes clade, fish from Rifle River (Lake Huron dr.) and Fleming Creek (Lake Erie dr.), Michigan diverged 0–12.3 ka (0%–0.01%), and these populations likely separated from fish now in Sodus Creek (Lake Ontario dr.), New York around 178–445 ka (0.2%–0.5%). In the Southeast clade, the Black Warrior River population (Mobile River dr.) diverged from the Dry Creek population (Tennessee River dr.) around 196–487 ka (0.2%–0.6%). In the Upper Midwest clade, the four populations diverged from each other more recently (0–36.3 ka), reflecting Pleistocene glacial dynamics.

### 3.2 Species Distribution Models

#### 3.2.1 MaxEnt Model Performance

For all time steps, each set of 10 model replicates reached an average training AUC of 0.7553 and a test AUC of 0.7551, indicating good performance. Percent permutation importance results indicated temperature annual range (Bio 7) was the most influential predictor of *R. obtusus* presence, followed by annual precipitation (Bio 12) and maximum temperature of the warmest month (Bio 5). These models represent an initial test of likely species presence in the past and present. While model parameters could be further optimized, these preliminary results provide a useful visualization of potential habitat suitability patterns for *R. obtusus*. Maxent model raster results are included in Supporting GIS Materials.

#### 3.2.2 Predicted Historical Distributions and Refugial Areas

**FIGURE 4:**
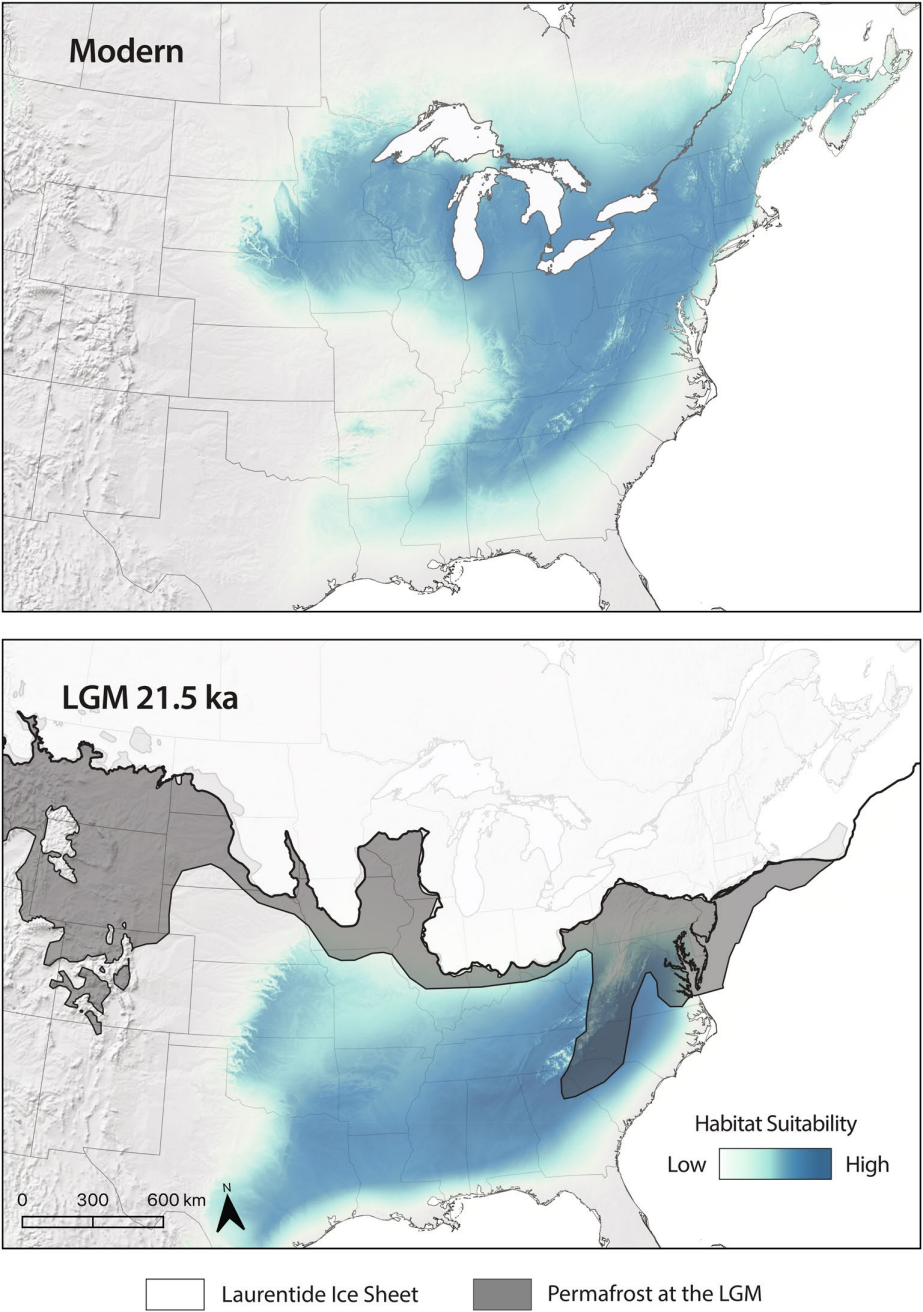
Geographic projections of species distribution models for the present‐day climate and the LGM, estimated using MaxEnt. The estimated LIS maximum extent (Dalton et al. 2023) and estimated permafrost extent (Lindgren et al. 2016) at 21.5 ka are illustrated, with overlap indicating slight discrepancies.

When projected to the LGM, the model predicted suitable habitat broadly throughout southeastern North America, with the highest suitability extending from the lower Central Highlands to the Atlantic Slope (i.e., Atlantic Gulf Coastal Plain) (Figure 4). This corresponds to well‐known refugial areas for freshwater fishes in the Mississippi Basin during the Pleistocene glaciations (Bailey and Smith 1981; Mayden 1988). It is important to note that although Atlantic Slope habitats were modeled as suitable, modern *R. obtusus* populations are only found isolated in three regions across these habitats (the Santee system in the Carolinas, the Savannah River basin in South Carolina and Georgia, and the upper Coosa River and Black Warrior Rivers of the Mobile Bay Basin), suggesting there was actually limited dispersal into these drainages followed by post‐glacial isolation. Therefore, the Central Highlands region was the most likely area of refuge for *R. obtusus* , with suitable habitat extending to just below the predicted permafrost extent (from Lindgren et al. 2016).

**FIGURE 5:**
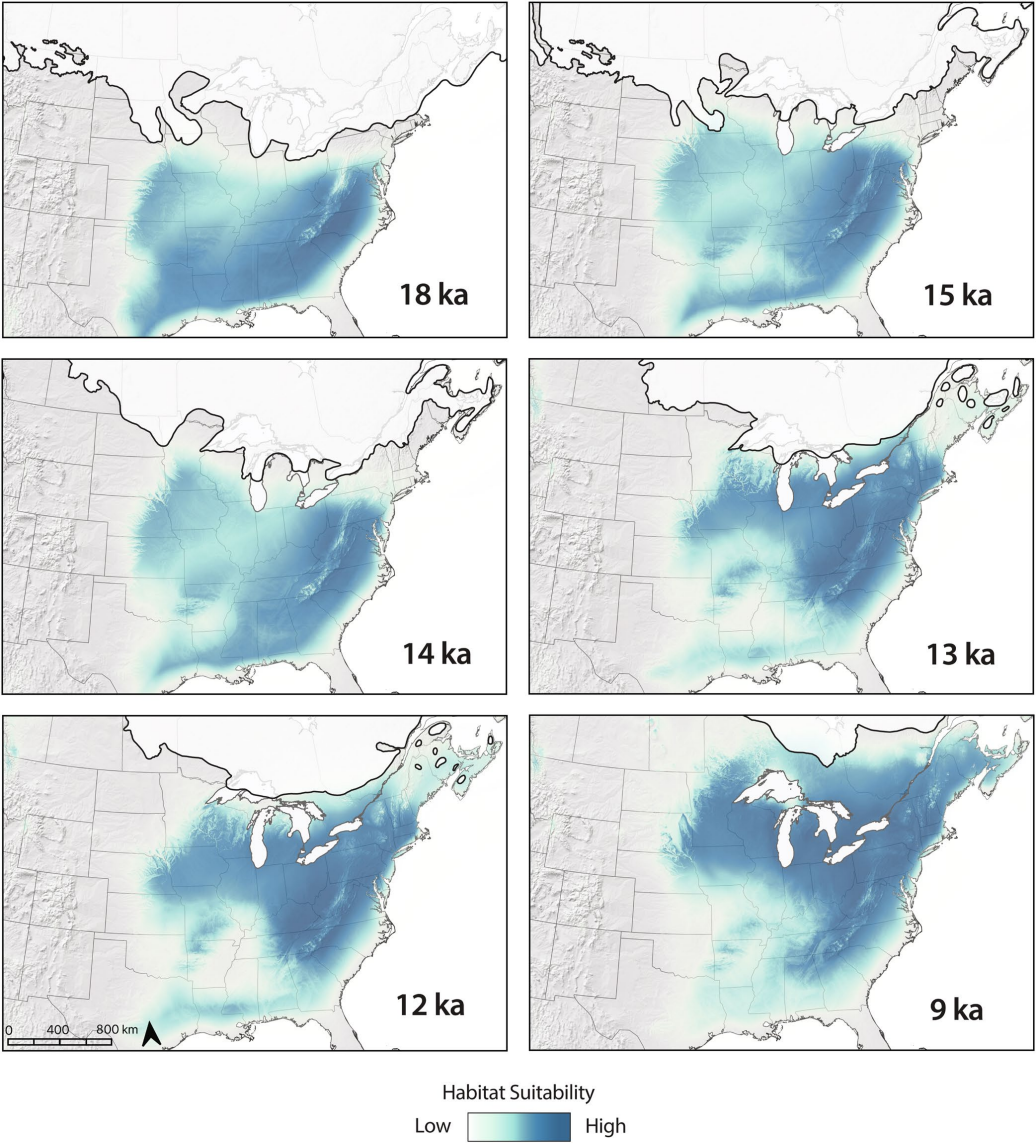
Geographic projections of species distribution models estimated using MaxEnt for six time steps after the LGM: 18, 15, 14, 13, 12, and 9 ka. The approximate maximum extents of the LIS are shown at each time step (Dalton et al. 2023).

Following the LGM (~21.5 ka), the Erie and Mackinaw periods of Laurentide Ice Sheet (LIS) retreat (19.2–18.2 ka and 16.1–15.6 ka) facilitated suitable habitats northward for recolonization of species (Bailey and Smith 1981; Wickert et al. 2023) (Figure 5). Around 15 ka, the model predicted highest habitat suitability in the Appalachian Plateaus (Spotila and Prince 2022), while the upper part of Lower Michigan and Lake Ontario were not yet accessible or suitable. Throughout the Port Huron readvance from 15.6–14.1 ka, the Two Creeks Retreat from 14.1 to 13.5 ka, and the Onaway advance of 13.5–12.9, the Lower Great Lakes were predicted to be relatively suitable for recolonization (Wickert et al. 2023). During the Younger Dryas (Gribben) period (12.9–11.7 ka), ice retreat continued and did not reverse northward range expansion, despite significant cooling. The LIS continued to retreat into Northern Canada from 12–9 ka and climate conditions became more optimal in the Great Lakes region and Upper Midwest regions.

## 4 Discussion

### 4.1 Glacial Refugial Areas and General Post‐Glacial Dispersal Pathways

**FIGURE 6:**
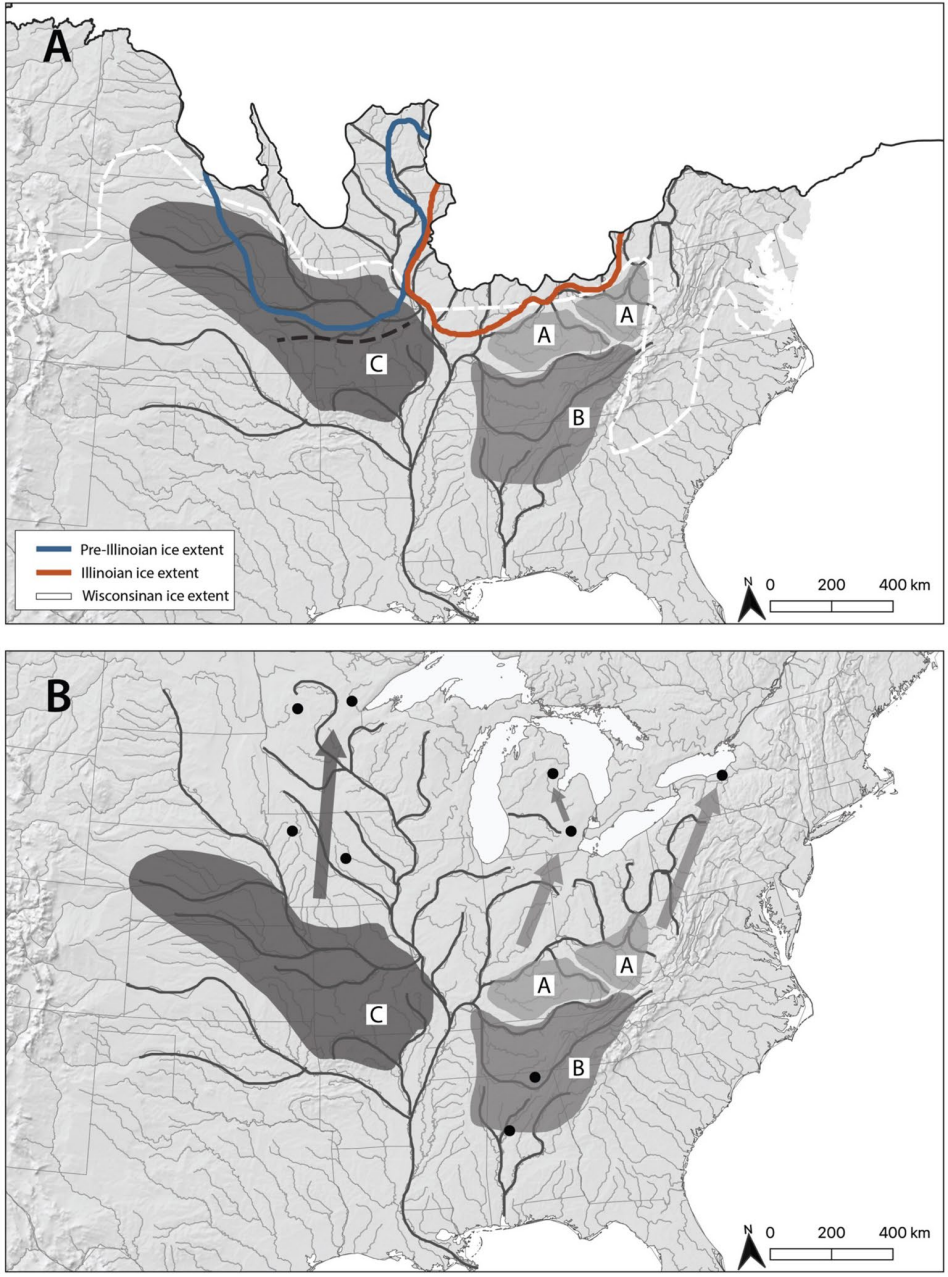
Proposed glacial refugial areas and dispersal pathways of *R. obtusus*. (A) Potential refugial areas for each of three major clades: A = Eastern Great Lakes clade, B = Southeast clade, C = Upper Midwest clade. Blue and red boundaries represent approximate Pre‐Illinoian and Illinoian glacial boundary extents, respectively (Burr and Page 1986, Schilling et al. 2013). The dashed white line represents approximate permafrost extent of the Wisconsinan (Lindgren et al. 2016). During the Pre‐Illinoian glacial maximum, the refugial area for clade C was likely no further than the dashed black line (and likely not in the Driftless Area). (B) Arrows indicate hypothesized interglacial and post‐glacial generalized dispersal pathways taken by the Eastern Great Lakes clade and Upper Midwest clade.

The recovery of an Upper Midwest clade and an Eastern Great Lakes/Southeast clade, diverging ~1.7–2.7 ma with 1.8%–3.2% sequence divergence (Figure 2, Figure 3), supports the Central Highlands pre‐Pleistocene vicariance event hypothesis (Mayden 1985, 1988; Wiley and Mayden 1985). This suggests that fish lineages with disjunct distributions in the Central Highlands and Upper Midwest previously had widespread distributions that were fragmented by the Pleistocene glaciations (Mayden 1985, 1988; Wiley and Mayden 1985; Burr and Page 1986; Near et al. 2001). The onset of the Pre‐Illinoian glaciation pushed *R. obtusus* southward, isolating populations in refugia in the Interior and Eastern Highlands (Figure 6A). While our sample sites are geographically broad and numerically limited, there is enough data to provide an initial test of general biogeographic hypotheses and establish a foundation for future phylogeographic work on this species.

Prior to the Pleistocene, the Central Highlands (now referred to as the Central Lowlands due to glacial reshaping) extended continuously across Ohio, Indiana, Illinois, Missouri, and Iowa, with topography and stream gradients similar to the modern‐day, unglaciated regions (Thornbury 1965; Cross et al. 1986; Bossu et al. 2013). Many freshwater fishes had continuous distributions across the landscape (Mayden 1985, 1988). The pre‐Pleistocene Teays‐Mahomet Valley system connected drainages from North Carolina westward to the Mississippi River, allowing for fish dispersal (Wayne 1952; Bossu et al. 2013). Advancing ice sheets disrupted the Teays‐Mahomet, as the eastern Teays River was diverted south into the Wabash and Ohio Rivers, while the western Mahomet River continued to flow into the Illinois River and then into the Mississippi (Wayne 1952; Robison 1986; Mayden 1988). Fish populations distributed throughout the Teays‐Mahomet system were pushed into refugial tributaries in the Central Highlands. The Pre‐Illinoian (~2.5–0.2 ma) ice sheet reached its maximum at the edge of the present‐day Missouri River and Ohio River, dividing the Central Highlands region into the Eastern and Interior Highlands on either side of the Mississippi River (Burr and Page 1986; Schilling et al. 2013). Later Pleistocene glaciations (Illinoian and Wisconsinan) further eroded and reshaped the Central Lowlands region north of these highlands.

Therefore, this vicariance event likely separated ancestral *R. obtusus* populations prior to 1.5–2.7 ma, where the Upper Midwest clade survived in the Interior Highlands and the Eastern Great Lakes and Southeast clades were pushed into the Eastern Highlands. We speculate populations of *R. obtusus* did not survive in the Driftless Area of Wisconsin while permafrost was extensive throughout the area until permafrost retreated, between approximately 19.4–4.7 ka (Clayton et al. 2001; French and Millar 2014; Carson et al. 2019; Schaetzl et al. 2022). The split between the Eastern Great Lakes clade and the Southeast clade ~1.5 ma likely resulted from the capture of the Lower Tennessee River by the Ohio River, diverting the Tennessee's course northward to join the Ohio River rather than southwest toward the Mississippi River during the mid‐Pleistocene (Mills and Kaye 2001; Galloway et al. 2011; Hoagstrom et al. 2014). This would have facilitated the transfer of *R. obtusus* from Ohio River refugia into the Tennessee River drainage and eventually into the Mobile Bay Basin. Any populations that already resided in or originated in the Atlantic Slope likely remained in the upper reaches of the Mobile Bay Basin and Apalachicola River Basin, with some populations later becoming isolated in the Savannah River and Santee River after the headwaters of the Chattahoochee River were captured by the Savannah River and diverted southeast during the Pleistocene (Swift et al. 1986; Bermingham and Avise 1986).

During interglacial cycles, populations likely expanded their ranges from respective refugia (Figure 6B). Similar patterns are observed in other freshwater fish taxa with disjunct distributions across North American drainages, including *Miniellus nubilus* (Forbes, 1878), *Erimystax dissimilis*, *Percina* subgenus *Odontopholis*, *Etheostoma* subgenus *Litocara*, *Etheostoma caeruleum* , *Chrosomus erythrogaster* , *Percina evides* , *Micropterus dolomieu* , and members of the *Macrhybopsis aestivalis* complex (Burr and Page 1986; Mayden 1988; Strange and Burr 1997; Near et al. 2001; Borden and Krebs 2009; Berendzen et al. 2010; Hoagstrom and Echelle 2022).

### 4.2 Biogeography of the Eastern Great Lakes Clade

**FIGURE 7:**
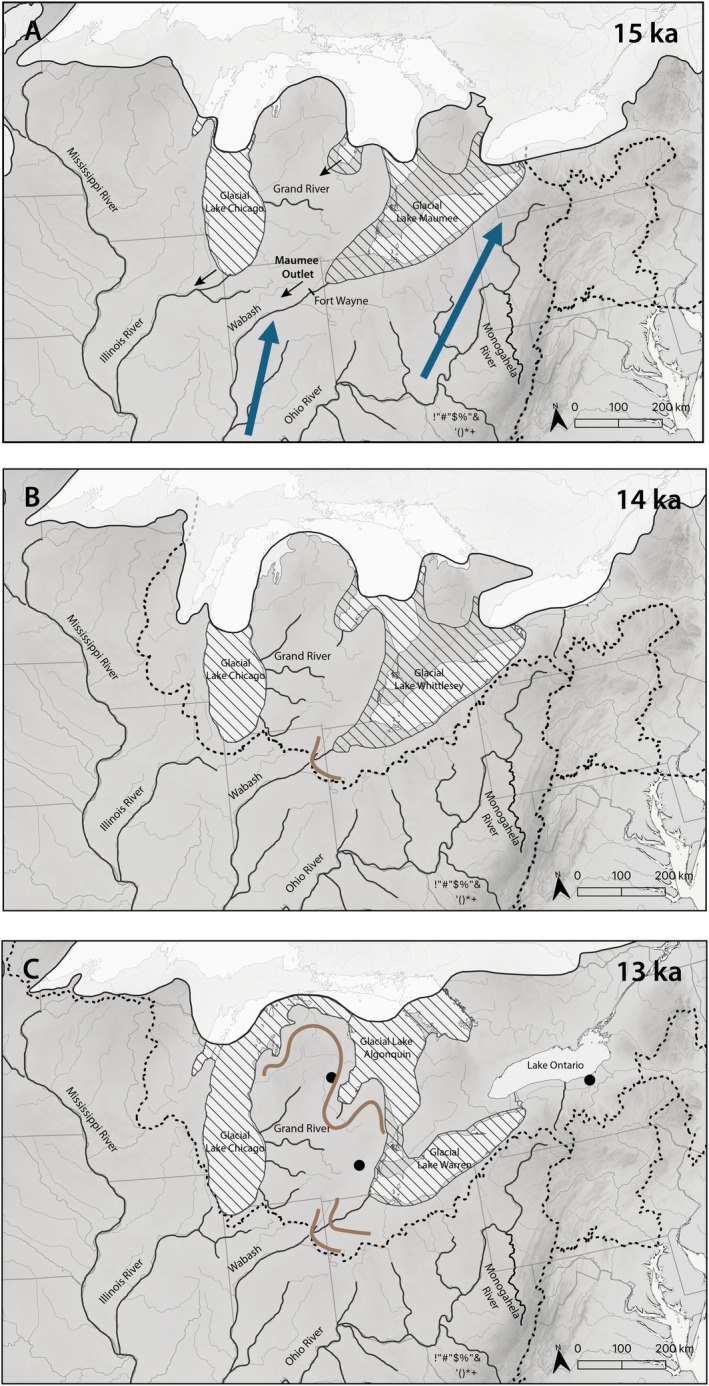
Hypothesized *R. obtusus* recolonization routes into the Eastern Great Lakes region by Eastern Great Lakes clade ancestral populations. (A) From around 17 ka to 14 ka, the Maumee Outlet, overflowing from Glacial Lake Maumee in the Erie basin to the Wabash River, may have been open as a dispersal pathway into Michigan. (B) By the end of the Port Huron Advance in 14 ka, the Maumee Outlet closed, and isostatic rebound led to the formation of the Lake Michigan and Lake Erie drainage basin boundaries (Bailey and Smith 1981; Wickert 2016). (C) The LIS retreated by 13 ka, allowing recolonization into Northern Lower Michigan, Southern Lower Michigan, and the Lake Ontario drainage. Brown lines represent prominent glacial moraines formed from decreasing proglacial lake levels and retreating ice (Hocutt and Wiley 1986; Kelley and Farrand 1988; Teller 2003; Krist and Lusch 2004; Sunderman et al. 2010; Wickert 2016; Dalton et al. 2023).

Our analyses suggest that populations of *R. obtusus* in Michigan entered Eastern Great Lakes drainages ~15–13 ka from Ohio River refugia. Around 17 ka, Glacial Lake Maumee (in place of modern‐day Lake Erie) was at its highest (800 ft) and overflowed at Fort Wayne in Indiana into the Wabash River Valley, periodically flooding the valley (Figure 7A) (Fleming et al. 2018). Subsequent Glacial Lake Whittlesey continued to drain into the Wabash River for approximately 2000 years, eventually closing due to crustal rebound (Bailey and Smith 1981; Wickert 2016; Wickert et al. 2023). This connection between the Maumee River outlet and Wabash River was undoubtedly a pathway many aquatic organisms took into Michigan and Lakes Huron and Erie (Figure 7A) (Bailey and Smith 1981; Mandrak and Crossman 1992; Teller 2003; Borden and Krebs 2009; Hewitt et al. 2018). This included larger fishes such as *Micropterus dolomieu* , the Smallmouth Bass, and smaller species such as *Etheostoma caeruleum* , Rainbow Darters, *Etheostoma flabellare* , Fantail Darters, and members of the genus *Notropis*, Shiners (Gerking 1947; Bailey and Smith 1981; Ray et al. 2006; Borden and Krebs 2009).

Continued ice retreat and overflow may have allowed access to minor stream pathways and connections in nearby floodplains, facilitating movement for small minnows such as *R. obtusus* . Although connections have not been confirmed geologically, adjacent tributaries of the Illinois River, St. Joseph River, and Wabash River may have been connected during periods of flooding (Gerking 1947). Additionally, the Port Huron readvance from 15.6 ka to 14.1 ka caused some overflow back into the Mississippi Basin, likely allowing passage into Michigan via streams or wetland habitats (Larson and Schaetzl 2001; Teller 2003; Wickert et al. 2023).

By 14 ka, water levels in Glacial Lake Warren in the Erie basin dropped, and the Port Huron end moraine system south and southwest of Lake Erie may have become a barrier to movement (Figure 7B) (Kelley and Farrand 1988; Larson and Schaetzl 2001; Wickert 2016; updated 2025, pers. comm.). *Rhinichthys obtusus* populations entered the Huron‐Erie lowlands after water levels in glacial lakes of the Erie basin (Maumee, Arkona, Whittlesey, Warren, Wayne, Grassmere, Lundy, and Rouge) dropped continuously, forming smaller moraines in a series of successive sediment deposits (Howard 2010). Recolonization of the Rifle River and other drainages in Central Lower Michigan likely occurred after 13 ka when climate conditions were more suitable after the LIS retreated and water levels in Glacial Lake Algonquin (in place of modern‐day Lake Huron) dropped sufficiently to allow a migratory pathway (Figure 7C) (Larsen 1988). This is supported by the divergence of Fleming Creek and Rifle River populations ~4 ka (0–12.3 ka) (Figure 3).

The Eastern Great Lakes clade also includes *R. obtusus* from Sodus Creek, NY (Lake Ontario basin), which diverged from Michigan populations ~300 ka (178–445 ka) (Figure 3). Admixture analysis of genome‐wide SNPs in these populations indicated very little gene flow or sharing of alleles between Sodus Creek and Michigan populations (Rodriguez 2025). Therefore, during the Pleistocene Illinoian and Wisconsinan glacial periods, this lineage may have persisted in a separate refugium in the southeastern portion of the Old Teays‐Mahomet system, potentially in the Kanawha region of the Appalachian Plateaus where climate conditions were favorable following the LGM (Figure 4).

When the Teays River was blocked and rerouted during the Pre‐Illinoian (2.5–0.3 ma), remnant tributaries, including the Kentucky and Licking Rivers and the Kanawha and New River systems, were integrated into the Ohio River drainage (Hocutt and Wiley 1986). Northeastward dispersal into the Lake Ontario basin ~13 ka would have been facilitated by shifting drainages, such as the growth of the Greenbrier River and its capture of the Upper Cheat River in the Monongahela drainage (Hocutt and Wiley 1986). Additionally, the Cuba Outlet connected the Upper Ohio and Monongahela River systems to the Genesee River that drains into Lake Ontario (Oswald et al. 2020; Dalton et al. 2023). Similar routes have been hypothesized for other taxa, including an *Etheostoma caeruleum* clade, a *Notropis rubellus* clade, and *Exoglossum laurae* (Ray et al. 2006; Berendzen et al. 2008; Oswald et al. 2020). This hypothesis is also supported by the presence of *R. obtusus* in the Gee Lick Run of the West Fork River in the Monongahela River Drainage, Big Laurel Creek of Williams River in the Kanawha River Drainage, and Four Mile Creek of the Allegheny River Drainage, which are related to a hybrid population of *R. obtusus* in Mudge Creek of the Lake Ontario Drainage (see figures 1 and 3 from Kraczkowski and Chernoff 2014). Furthermore, Hubbs and Lagler (1958; see also the revised version Hubbs et al. 2004) speculated that members of the *Rhinichthys atratulus* complex were also present in the Monongahela, eventually moving eastward into the Potomac.

Alternatively, Sodus Creek fish could have persisted in refugia nearby ancestral Michigan populations in the Wabash River, Green River, or western Ohio River, then entered Michigan around the same time. Dispersal to western Lake Ontario could have occurred around the lower edge of Lake Erie through various stream pathways, or above Lake Erie to reach Sodus Creek prior to the formation of Niagara Falls at 12.5 ka (Mandrak and Crossman 1992).

### 4.3 Biogeography of the Southeast Clade

**FIGURE 8:**
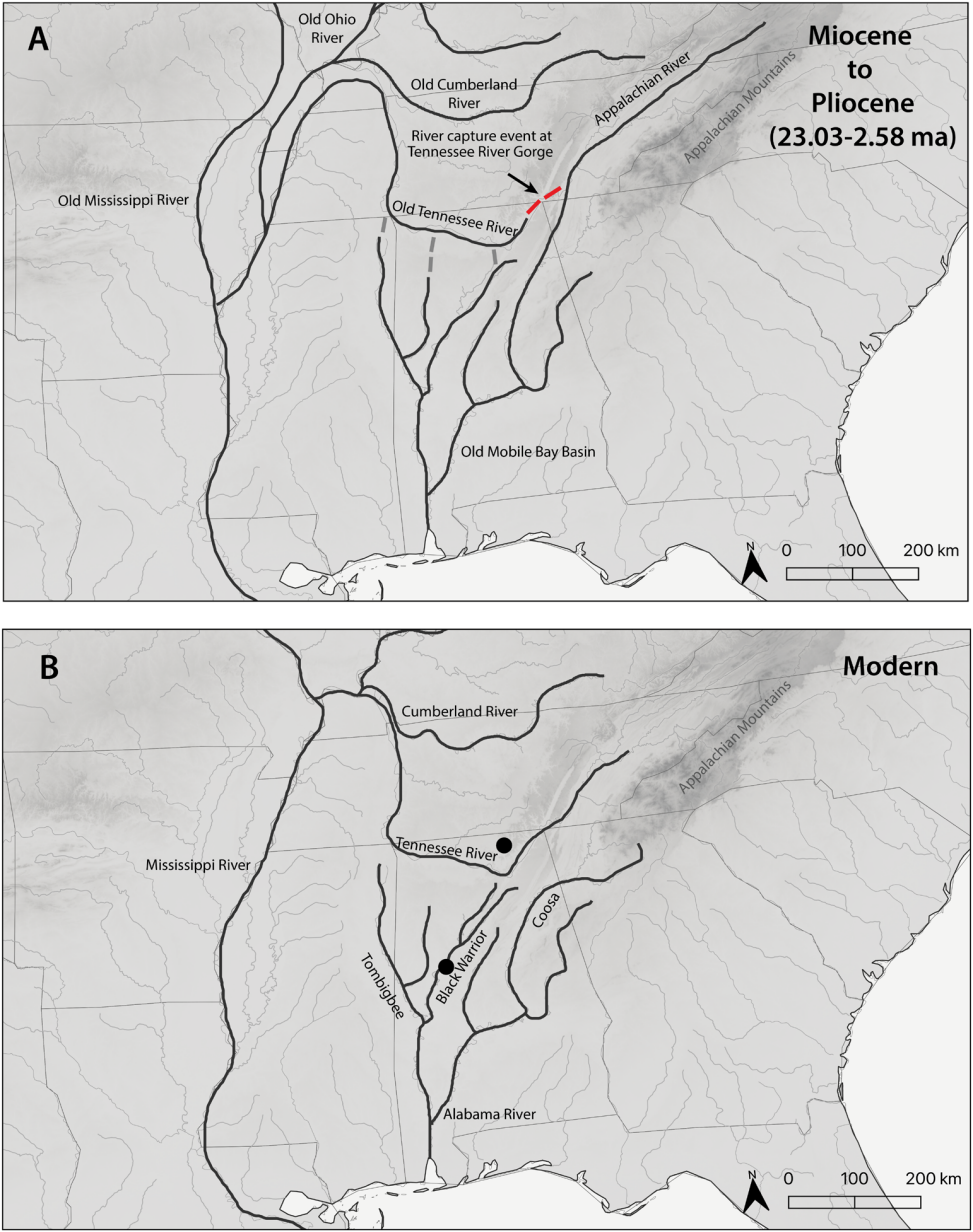
Historical drainage connections between the Tennessee River and the Mobile Bay Basin and present‐day separate drainages showing a potential vicariance split in populations. (A) River reconstructions of the Old Mobile Bay Basin, Old Mississippi drainages, and proposed Paleo Appalachian River during the Miocene and Pliocene based on Burr and Page (1986), Mayden (1988), and Odom and Granger (2022). The Paleo Appalachian River may have flowed into the Coosa River before the Middle Tennessee River diverted it west at the Tennessee River Gorge (red dashed line) (Hayes and Campbell 1894; Swift et al. 1986; Mayden 1988). The light gray dashed lines represent different speculated connections of the Tennessee to the Mobile Bay Basin (Hayes and Campbell 1894; Swift et al. 1986; Mayden 1988; Mills and Kaye 2001; Wood et al. 2021; Odom and Granger 2022). (B) Modern Tennessee River drainage and Mobile Bay Basin. The black dots represent our two Alabama populations, at Dry Creek in the Tennessee River drainage and at Black Warrior River in the Mobile Bay Basin.

The *R. obtusus* populations from Dry Creek (Middle Tennessee River) and the Black Warrior River (Mobile Bay Basin) represent an important phylogeographic break associated with the geological separation between these two major drainage basins (Mayden 1988). Currently, the Tennessee River flows north to meet the Ohio River just before joining the Mississippi River, while the Black Warrior River drains south into the Mobile Bay and then to the Gulf of Mexico (Figure 8B). These drainages are no longer connected, but the presence of closely related fish lineages in both drainages may suggest that previous connections existed (Swift et al. 1986; Mayden 1988; Wood 1996; Fluker et al. 2019).

The geomorphological history of the Tennessee River has been debated in the literature for over a century. While the Tennessee River's present‐day course is U‐shaped, there are Late Cretaceous to Eocene‐aged fluvial deposits near Mobile, AL that indicate the Old Tennessee River likely flowed through the Mobile Bay Basin into the Gulf of Mexico (Blum et al. 2017; Odom and Granger 2022). The precise course and location of connections between drainage basins, as well as the separation events, remain unresolved due to limited geologic evidence.

Some sources have proposed an ancient “Appalachian River” that ran from the Appalachian Mountains (where the Upper Tennessee River is now) south into the old Mobile Basin through the Coosa River into the Gulf of Mexico (Figure 8A) (Hayes and Campbell 1894; Swift et al. 1986; Mayden 1988). A river capture event at the Tennessee River Gorge may have occurred during the Miocene due to the lower stream gradient of the Upper Tennessee, facilitating the separation and transfer of fish fauna between these drainages (Mayden 1988). Rather than a larger Appalachian River and single vicariance split, Mills and Kaye (2001) noted gravel deposits along the Tombigbee, Sipsey, and Black Warrior Rivers that could indicate smaller connections to the Tennessee. However, the deposits are poorly preserved and there is no clear evidence of a main channel (Starnes and Etnier 1986; Russell and Schmitz 2003; Odom and Granger 2022). Alternatively, local headwater capture events between the Tennessee River and Mobile Basin rivers, such as Graves Creek of the Locust Fork which connects to the Black Warrior River (Wood et al. 2021), could have allowed for faunal transfer at various points (Figure 8A).

Despite limited geologic evidence, phylogeographic data strongly suggest a historical link. Swift et al. (1986) proposed two periods of connections in the early‐ and late Tertiary, based on evidence from numerous upland Tennessee species that have close relatives in the Mobile Bay Basin, including species belonging to *Notropis*, *Hemitremia, Moxostoma*, and *Campostoma*. Furthermore, Mayden (1988) noted that fish fauna from the Mobile Bay Basin form sister groups to all Mississippi drainage rivers. Tertiary connections are also supported by Near and Keck (2005), who found that *Nothonotus* darter populations in the lower Tennessee River and Mobile basin rivers diverged 9.0 ± 1.7 ma during the mid‐Miocene. Similarly, Jones et al. (2006) found a break in lineages no earlier than 10 ma. A decrease in sediment deposits in the Gulf from the Mobile basin around this time may also correlate with a break in the drainages (Blum et al. 2017).

Interestingly, Bayesian analysis of full mitochondrial genome sequences suggested that our AL populations diverged much more recently, around 196–487 ka during the Pre‐Illinoian glaciation (Figure 3). We speculate that there may have been a more recent Pleistocene connection, or connections, between tributaries of the Tennessee River and the Mobile Bay Basin. One possibility is that Pleistocene glacial meltwater and sedimentary obstructions led to spillover of water from the Tennessee River into the Black Belt Prairie of Mississippi, which overlies the Mobile Bay drainage (Kaye 1974), allowing movement between drainages. Due to the close proximity of upland headwaters in the Mobile basin to the Tennessee River, it seems likely that small stream connections could have appeared and disappeared during the Pleistocene. Another possibility is that *R. obtusus* transferred basins through subterranean pathways in karst cave systems, as proposed for *Chrosomus erythrogaster* , the Southern Redbelly Dace (Ray et al. 2014).

### 4.4 Biogeography of the Upper Midwest Clade

We propose that the Upper Midwest clade of *R. obtusus* dispersed north from glacial refugia west of the Mississippi River in the late‐Pleistocene. Among freshwater fish taxa more broadly, phylogeographic patterns indicate a close relationship between drainages of the Upper Midwest and Interior Highlands (Ozark and Ouachita Plateaus). This corresponds to the general post‐glacial recolonization of northern areas from western refugia, rather than a recent east‐to‐west dispersal from the Eastern Highlands. Other freshwater species that contain closely related lineages in these two regions include *Miniellus nubilus* (Mayden 1988), *Hypentelium nigricans* (Berendzen et al. 2003), *Notropis percobromus* (Berendzen et al. 2008), and *Percina evides* (Near et al. 2001).

**FIGURE 9:**
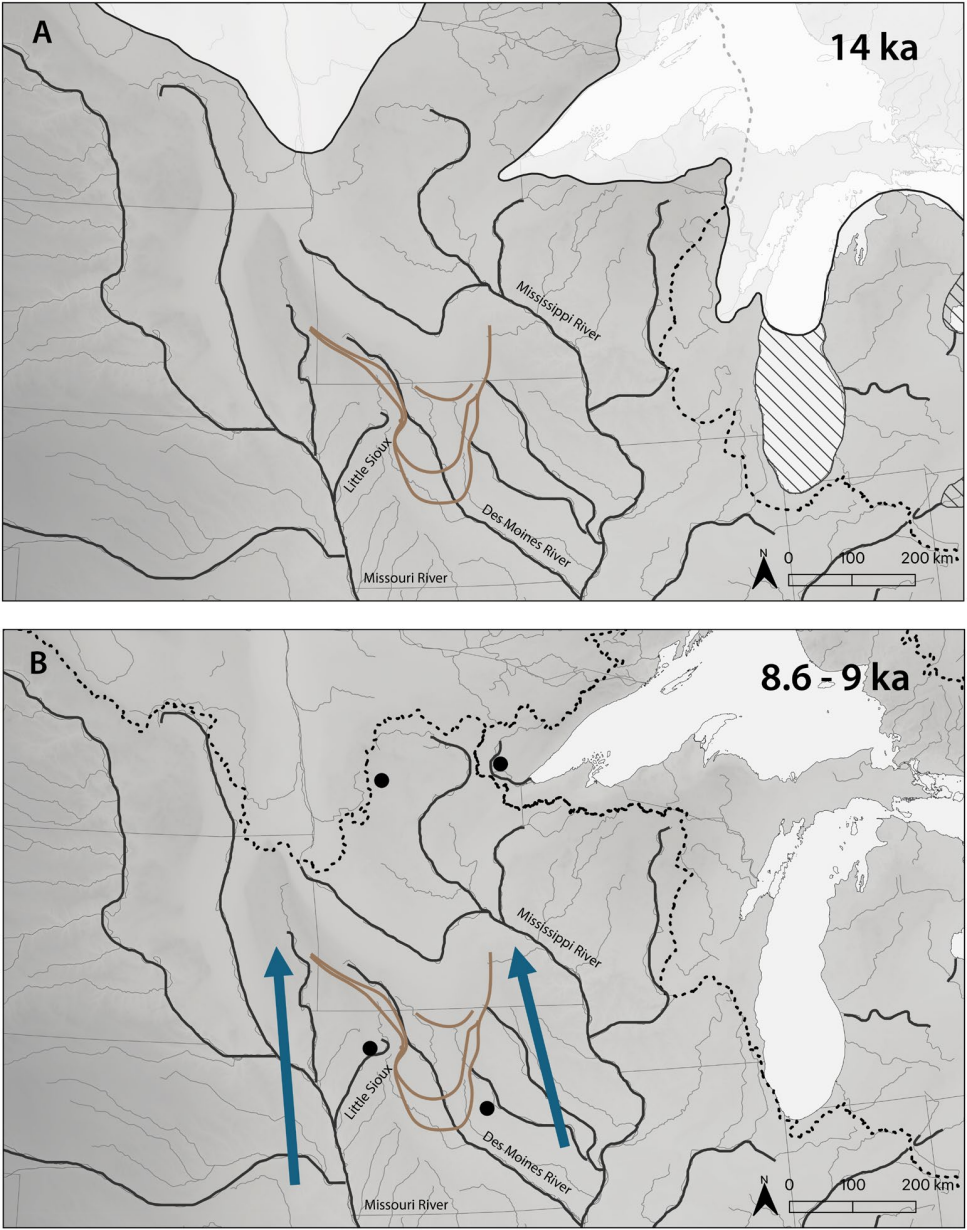
Reconstructed biogeographic scenarios illustrating the hypothesized recolonization of *R. obtusus* into the Upper Midwest region. This likely occurred through various dispersal and faunal transfer events, after 14 ka during periods of river connectivity, flooding, or stream capture. (A) The LIS retreated out of the Upper Midwest and Missouri River drainages after the Port Huron Advance from 15.2–14 ka (Wickert et al. 2023). The deposition of moraines after the LGM, represented by the brown lines, may have facilitated the separation of populations in the Des Moines River drainage and the Little Sioux drainage. (B) The larger St. Lawrence River drainage boundary and the Lake Superior drainage formed by 8.6–9 ka, suggesting the Spider Creek population likely entered the Lake Superior drainage by then (Cross et al. 1986; Bailey and Allum 1962; Wickert 2016; Dalton et al. 2023).

In general, northward migration of *R. obtusus* to the Upper Midwest occurred during the late‐Pleistocene when ice had melted and drainages in the Upper Midwest and Lake Superior were relatively connected (Figure 9A). During the Pre‐Illinoian glaciation, refugial areas were likely confined to the northern Interior Highlands south of the Missouri River, below the extent of the ice sheet (Figure 6A). By the Illinoian and Wisconsinan stages, ice margins reached only central Iowa, and refugial areas may have expanded northeast throughout the Old‐Grand Missouri River system. These glacial fluctuations and shifting suitable habitat for refugial areas likely facilitated gene flow and mixing between populations during the Pleistocene. This is demonstrated for *R. obtusus* Upper Midwest populations by short branch lengths and unresolved node placement in both the Maximum‐Likelihood tree and BEAST2 Bayesian analysis (Figures 2, 3), indicating close phylogenetic relationships and recent divergence. Recolonization of northern drainages also resulted in founder effects and reduced genetic diversity (Adhikari et al. 2025).

It was likely a combination of events at different times that facilitated movement to various drainages in the Upper Midwest. Following the LGM, melting ice gave way to episodic flooding and river capture events, and to various drainage connections which would have allowed movement in multiple directions (Bailey and Allum 1962). Connections occurred between the Minnesota and Des Moines River of the Upper Midwest region and the Big Sioux and Little Sioux Rivers that are tributaries to the Missouri River (Bailey and Allum 1962; Cross et al. 1986). In fact, the deposition of glacial moraines and loess in central Iowa (Mayden 1985; Arthur Bettis III et al. 2003; Muhs 2007; Dalton et al. 2023) may reflect a post‐LGM separation event between the ancestral Waterman Creek population in the Little Sioux watershed from the ancestral Minerva Creek population in the Iowa River watershed (Figure 9B). Other species, including *Pimephales notatus* and *Miniellus stramineus*, may have moved in the opposite direction southward into the Missouri basin from rivers in the Upper Mississippi system (Bailey and Allum 1962).

Furthermore, the modern absence of *R. obtusus* south of the Missouri River in the Interior Highlands may reflect an extirpation event in ancestral *R. obtusus* populations as a result of habitats becoming unsuitable. This is supported geologically by glacial loess deposits concentrated in Iowa, Missouri, Nebraska, and Kansas that transformed suitable high‐gradient, rocky streams into unsuitable turbid, soft‐sediment streams in the area (Mayden 1985; Arthur Bettis III et al. 2003; Muhs 2007). Overall, this could have driven *R. obtusus* populations northward.

## 5 Conclusion

The phylogeographic history of *R. obtusus* reflects the combined influences of the Pleistocene glaciations, large‐scale shifts in drainages, and stream and river capture events that shaped the species' distribution and mitochondrial genomic landscape. We identified three major clades: Eastern Great Lakes, Southeast, and Upper Midwest. These clades likely diverged during a major vicariance event at the onset of the Pleistocene, when the Pre‐Illinoian ice sheet fragmented the ancient Teays‐Mahomet River and separated its formerly continuous distribution across the region. Populations in the Eastern Great Lakes clade likely persisted in multiple Ohio River refugia before dispersing northward into Michigan and, via stream capture events, into Southeastern Lake Ontario drainages. The mid‐Pleistocene capture of the Tennessee River by the Ohio River may have facilitated faunal transfer into the Tennessee drainage and Mobile Bay Basin, likely driving divergence of the Southeast clade from the Eastern Great Lakes clade. The Upper Midwest clade likely descended from populations that survived in refugia west of the Mississippi in the Interior Highlands and Missouri River system, dispersing northward after approximately 14 ka when glacial loess deposits began transforming the landscape. Future work incorporating expanded sampling and genomic data may help resolve the nuclear genomic and genetic evolution of *R. obtusus* over time. With improvements in sequencing technology and DNA data availability, as well as higher‐quality paleoclimate and geologic data, there are greater opportunities for integrative research in the field of comparative phylogeography of North American freshwater fish.

## Author Contributions

**Adelina Rodriguez:** conceptualization (lead), data curation (lead), formal analysis (lead), investigation (lead), methodology (lead), project administration (lead), resources (lead), software (lead), supervision (lead), validation (lead), visualization (lead), writing – original draft (lead), writing – review and editing (lead). **Timothy S. Earley:** conceptualization (supporting), data curation (supporting), software (supporting), validation (supporting), writing – original draft (supporting), writing – review and editing (supporting). **Samuel Taylor:** conceptualization (supporting), writing – review and editing (supporting). **Thomas E. Dowling:** writing – review and editing (supporting). **Phillip M. Harris:** writing – review and editing (supporting). **Jeremy Wright:** writing – review and editing (supporting). **Antonio Machado‐Allison:** conceptualization (supporting), writing – review and editing (supporting). **Barry Chernoff:** conceptualization (supporting), funding acquisition (lead), supervision (supporting), writing – original draft (supporting), writing – review and editing (supporting).

## Funding

This work was supported by Smith Funds from the Department of Earth & Environmental Sciences, Robert F. Schumann Foundation Bailey College of the Environment, Wesleyan University.

## Conflicts of Interest

The authors declare no conflicts of interest.

## Data Availability

Sample reads and data are available through NCBI BioProject PRJNA1260469, found at the reviewer link https://dataview.ncbi.nlm.nih.gov/object/PRJNA1260469?reviewer=ppc24nbllladjrloe3nttntc3b. Supporting Phylogenetic Materials and Supporting GIS Materials are available as part of a FigShare collection at https://doi.org/10.25438/wes02.c.8292601.
